# Comparison of Photocatalytic Performance of Sonochemically Synthesized ZnO with Different Capping Agents

**DOI:** 10.1021/acsomega.5c00929

**Published:** 2025-07-09

**Authors:** Tatiana Rodríguez-Flores, Isaías Hernández-Pérez, Gloria Elena de la Huerta-Hernández, Yadira Ayala-Parada, Jessica Guadalupe Cadena-Silva, Catalina Haro-Pérez

**Affiliations:** Departamento de Ciencias Básicas, 27786 Universidad Autónoma Metropolitana-Azcapotzalco, Av. San Pablo 420, C.P 02128 Ciudad de México, Mexico

## Abstract

This study investigates the influence of various organic modifiers that serve as capping agents on the structure, morphology, electronic properties, colloidal stability, and photocatalytic behavior of ZnO nanoparticles synthesized using the sonochemical method. The capping agents used include citric acid (CA), ethylene glycol (ETG), oleic acid (OA), and poly­(vinylpyrrolidone) (PVP). The synthesized ZnO nanoparticles were characterized using X-ray diffraction (XRD), scanning electron microscopy (SEM), transmission electron microscopy (TEM), energy dispersive X-ray Spectroscopy (EDS), diffuse reflectance ultraviolet–visible spectroscopy (DRUV–vis), dynamic light scattering (DLS), and electrophoresis (EP). XRD analysis confirmed that all samples exhibited a Wurtzite-type hexagonal structure, with crystal sizes ranging from 17 to 22 nm and band gap energies (*E*_g_) between 3.16 and 3.25 eV. Notably, samples synthesized with ETG and OA showed additional absorbance in the visible region. Among the samples, ZnO-ETG demonstrated the highest photodegradation efficiency for reactive black-5 azo-dye (RB5), serving as a model reaction. This work highlights the ability to modulate the properties of ZnO nanoparticles, such as crystal size, morphology, hydrodynamic size, surface charge and Urbach energy, through the choice of organic modifiers that serves as stabilizers, impacting their potential applications in photocatalysis.

## Graphical abstract

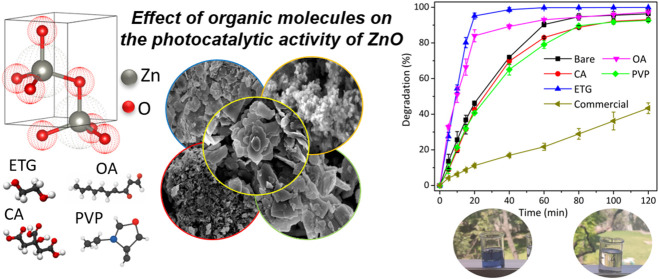

## Introduction

The exponential growth of the global population has resulted in overexploitation and contamination of natural resources. Water, a vital resource for sustaining life, has been significantly affected and has become one of the most severely polluted resources.^1,2^ The pollution of rivers and lakes can be primarily attributed to agricultural and industrial activities.^3,4^ Among various industries, the textile industry stands out as a major contributor to water pollution, largely due to the extensive use of highly concentrated dyes during the dyeing process.^5,6^ The chemical stability of these dyes poses significant challenges for their removal using conventional techniques such as filtration, coagulation, and sedimentation,^7^ which exhibit limited efficiency. Therefore, alternative methods that offer higher levels of dye degradation, such as photocatalysis, have been proposed. The most widely used photocatalyst is based on metal oxide semiconductors,^8,9^ zinc oxide (ZnO) is one of the most promising materials.^10–14^ However, ZnO’s activity is limited to the UV region, prompting extensive research to extend its efficacy to the visible region for wastewater contaminant removal. The use of surfactants as organic modifiers has emerged as a promising approach for synthesizing ZnO NPs with controlled parameters such as morphology, particle size, and optical properties, which directly impact their photocatalytic performance.^15–22^ Surfactant-assisted methods such as coprecipitation^21,23^ and sol–gel techniques^24,25^ have been employed to improve ZnO performance. However, few studies have explored the potential of sonochemical synthesis despite of being more efficient and sustainable compared to more traditional methods.^26^ Several researchers have demonstrated that the type and concentration of surfactants such as CTAB, SDS, PEG, and PVA influence ZnO’s crystallinity, optical properties, and photocatalytic behavior. For instance, SDS-modified ZnO synthesized via sonochemistry showed superior photodegradation of Rhodamine B under visible light.^21^ Similarly, El-Shazly et al.^22^ found that ZnO synthesized with CTAB exhibited the highest photocatalytic degradation efficiency of methylene blue (MB), attributed to reduced particle size and enhanced surface defects. Weldekirstos et al.^23^ also reported increased MB degradation in ZnO prepared with CTAB compared to EDTA and polyacrylamide, confirming the importance of surfactant type in shaping ZnO’s functional performance. Halvaeifard et al.^24^ and Kumar et al.^25^ demonstrated that cationic and nonionic surfactants like CTAB and PEG effectively modulate the band gap, surface energy, and morphology of ZnO particles, while Kiruthiga et al.^27^ highlighted the role of PVA in producing well-dispersed spherical ZnO nanoparticles with good optical activity. Other authors have proposed the use of Citrus extract as a stabilizing agent in the green synthesis of ZnO nanoparticles, demonstrating its applicability in the degradation of dyes such as methylene blue, rhodamine B, and methyl orange.^28^ Despite these contributions, most comparative studies are limited to two or three capping agents and primarily use conventional methods. Moreover, photocatalytic evaluations typically focus on MB under standard pH conditions and there is limited investigation into other relevant dyes such as reactive black 5 (RB5) under different pH conditions. Additionally, few reports simultaneously assess the structural, optical, colloidal stability, and photocatalytic properties of ZnO synthesized under uniform conditions with multiple organic modifiers. In this study, ZnO nanoparticles were synthesized using a sonochemical method with four different capping agents: citric acid (CA), ethylene glycol (ETG), oleic acid (OA), and poly­(vinylpyrrolidone) (PVP). We investigate how each molecule influences the crystal structure, optical band gap, colloidal stability, and surface morphology of the ZnO, and how these properties relate to the photocatalytic degradation of reactive black 5 (RB5) under varying pH conditions and methylene blue (MB) at natural pH under UV light.

In fact, the use of citric acid (CA), ethylene glycol (ETG), oleic acid (OA), and poly­(vinylpyrrolidone) (PVP) in the synthesis of materials other than ZnO has been shown to significantly enhance photocatalytic activity. For instance, PVP has been reported to improve dye degradation efficiency in BiOBr^29^ and Mn–CdS systems.^30^ ETG-assisted synthesis of Ag_8_SnS_6_ nanoparticles has also led to enhanced photocatalytic performance.^31^ Citric acid, used both as a surfactant and carbon source, enabled the formation of MoIn_2_S_4_ with carbon quantum dots with superior activity.^32^ Similarly, OA has had a positive impact on the photocatalytic behavior of CuO nanoplates^33^ and Cu_2_ZnSnS_4_ nanoparticles.^34^

## Experimental Section

### Materials

Nanocrystalline ZnO was synthesized via a sonochemical method using zinc nitrate hexahydrate (reagent grade, 98%) as the precursor. Four different organic compounds were employed in the synthesis: citric acid (CA, purity ≥99.5%), ethylene glycol (ETG, reagent plus, ≥99.0%), oleic acid (OA, technical grade, 90%), and poly­(vinylpyrrolidone) (PVP, molecular weight 40,000). Ethanol (ACS reagent, ≥99.50%) was used as the solvent, while a 4 M solution of sodium hydroxide (ACS reagent, ≥97.0%) served as the precipitant agent. All chemicals were supplied by Merck and used as received. For comparison, commercial ZnO nanopowder with a particle size of less than 50 nm (Product Number 677450) was also obtained from Merck.

### ZnO Synthesis

Bare-ZnO nanoparticles were synthesized by dissolving 14.4625 g of zinc nitrate hexahydrate in 50 mL of ethanol, being the precursor concentration of 0.97 M. The solution was then subjected to ultrasonication for 60 min (20 kHz, 90 W). Subsequently, 25 mL of a 4 M NaOH solution was added dropwise under ultrasonic irradiation until a white precipitate formed, a process that took over 15 min. The pH was monitored at the beginning, during, and at the end of the process, and remained constant at 12. ZnO synthesis is carried out at this pH because the elevated concentration of hydroxide ions (OH^–^) in alkaline media promotes their reaction with Zn^2+^ to form zinc hydroxide (Zn­(OH)_2_), which subsequently dehydrates to produce ZnO, ensuring a complete and uniform precipitation of the Zn precursor. Afterward, the precipitate was washed four times with deionized water. The resulting suspension was then filtered and dried at 70 °C for 24 h. To obtain ZnO in the presence of organic additives, the same protocol was followed, except the modifying agent was added to the alcoholic solution of zinc nitrate at a Zn/surfactant molar ratio of 0.05. The resulting dried ZnO nanoparticles were subsequently heat-treated at 350 °C for 2 h in static air. Thermal treatment is usually applied to enhance the crystallinity of the material, promoting the coalescence of smaller crystals into larger and more stable structures.^35^ Additionally, it facilitates the decomposition of the organic compounds used during synthesis, which should result in cleaner and purer ZnO surfaces. The synthesized samples were labeled as follows: bare-ZnO (without capping agent), ZnO-CA (with citric acid), ZnO-ETG (with ethylene glycol), ZnO-OA (with oleic acid), and ZnO-PVP (with poly­(vinylpyrrolidone)).

### Characterization Techniques

The samples were morphological and structural characterized by scanning electronic microscopy (Carl Zeiss SUPRA 55Pv), transmission electron microscopy (JEOL ARM-200F) and X-ray diffraction (XRD) measurements (X’Pert PRO Phillips, 40 kV, 35 mA, with a Cu Kα source). The crystallographic phases were identified based on the JCPDS 36–1451 standard and the crystal size was determined using the Debye–Scherrer equation

1 $$D=\frac{k\lambda }{\beta \left(\cos\theta \right)}$$

where *k* is a constant equal to 0.9, λ is the X-ray wavelength, β is the full-width at half-maximum of the diffraction peak corresponding to the (101) plane, and θ is the Bragg angle.

Elemental analysis by scanning transmission electron microsopy-energy dispersive X-ray spectroscopy (STEM-EDS) was also performed using a JEOL ARM-200F transmission electron microscope equipped with a field emission Schottky ZrO/W electron source. The microscope possesses a spatial resolution of 0.08 Å in STEM mode.

The ZnO ζ-potential and hydrodynamic diameter were determined using dynamic light scattering (DLS) with a Zetasizer Nano ZS90 instrument from Malvern Instruments. To perform the measurements, 1 mg of the sample was dispersed in 40 mL of ultrapure deionized water and subjected to ultrasonication using an ultrasonic tip (Ultrasonic processor FS-450N) operating at a frequency of 40 kHz and a power of 135 W for 8 min. The optical properties, including the band gap and Urbach energy, were calculated from Tauc’s and Urbach’s equations, respectively. These measurements were carried out using an ultraviolet–visible (UV–vis) spectrophotometer (Varian Cary IG) equipped with an integration sphere DRA-CA-30I, the photoluminescence spectrum was obtained in a Varian Cary Eclipse spectrofluorometer, equipped with a xenon lamp, the used excitation wavelength was of 370 nm. Finally, the Fourier transform infrared (FT-IR) measurements were carried out in a PerkinElmer FT-IR spectrometer (Frontier).

### Photocatalytic Degradation of Reactive Black 5 (RB5) and Methylene Blue (MB)

The photocatalytic degradation of RB5 and MB dyes was performed in a custom-made batch reactor equipped with a cooling jacket and subjected to continuous stirring. A 20 W UV lamp (Tecnolite), emitting light at a wavelength of 365 nm, was used as the irradiation source. A 250 mL of the dye solution was prepared at a concentration of 20 ppm, and 1.0 g/L of synthesized ZnO was added. To achieve the adsorption–desorption equilibrium, the dye solution containing the catalyst was kept in the dark and stirred continuously for 30 min. At desired time intervals, approximately 3 mL of the mixture was collected and filtered using an SFCA filter with a 0.2 μm pore size to separate the photocatalyst from the dye solution. The dye concentration was measured using a UV–vis spectrophotometer (Varian Cary IG). The degradation percentage was determined using the following equation

2 $$\%\mathrm{degradation}=\frac{A_{0}-A_{t}}{A_{0}}\times 100=\frac{C_{0}-C_{t}}{C_{0}}\times 100$$

where *A*_0_ represents the initial absorbance of the dye solution measured at the characteristic wavelength of each dye (597 nm for RB5 and 664 nm for MB) and *A_t_* is the absorbance of the solution at time *t*. The absorbance values were converted to concentrations, *C*, using calibration curves previously established for each dye.

## Results and Discussion

### Structural and Morphological Analysis

Based on the structural analysis shown in Figure 1, all the diffraction reflections can be indexed to the hexagonal ZnO Wurtzite type structure, regardless of the organic compound used in the synthesis. This observation is consistent with the JCPDS 36–1451 ZnO standard, indicating that the presence of organic additives in the synthesis does not alter the preferential orientation in the (101) plane of ZnO. Moreover, no discernible differences were observed in the diffraction patterns between the synthesized ZnO materials and the commercially available ZnO, as shown in Figure S1 of the Supporting Information (SI).

**Figure 1:**
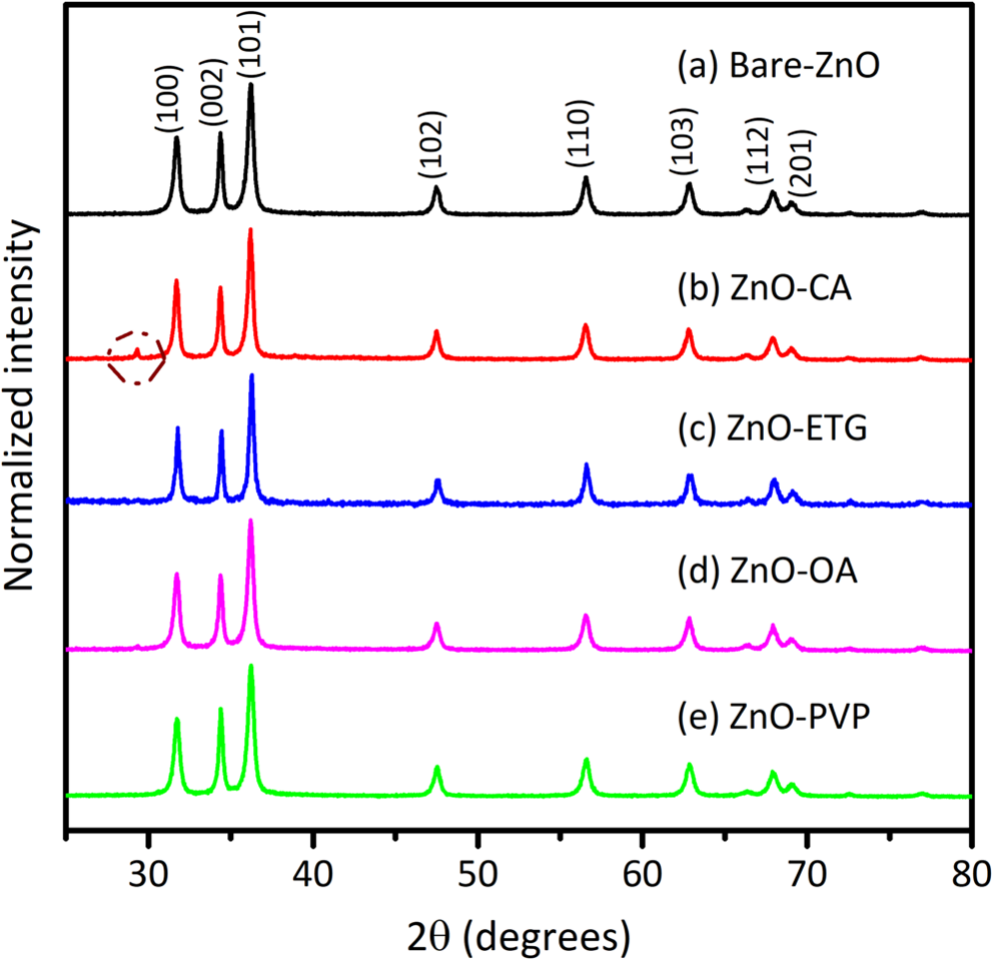
Diffraction patterns of ZnO synthesized without and with various capping agents; (a) Bare-ZnO, (b) ZnO-CA, (c) ZnO-ETG, (d) ZnO-OA and (e) ZnO-PVP.

The sample synthesized with citric acid exhibits a reflection around 30° (2θ), which corresponds to Zn­(OH)_2_ according to the letter JCPDS No. 38–0385, which is an intermediate in the formation of ZnO.^36,37^ However, no additional peaks corresponding to impurities were detected in the other samples. Furthermore, the crystal size of all the synthesized materials was determined using the Scherrer equation, considering the most intense peak located in the (101) plane as can see in Table 1.

**Table 1:** Crystal Size (nm), Lattice Parameters (Å) and Cell Volume (Å^3^) of ZnO Synthesized without and with Various Capping Agents, and for the Commercial ZnO

		cell parameters (Å)		
capping agent	crystal size (nm)	*A* (Å)	*C* (Å)	cell volume (Å^3^)
bare	17	3.2572	5.2154	48
CA	20	3.2582	5.2183	48
ETG	22	3.2512	5.2095	48
OA	18	3.2572	5.2154	48
PVP	17	3.2562	5.2154	48
commercial	23	3.2522	5.2495	48

Table 1 presents the dependence of crystal size on the organic modifier used during synthesis. Contrary to the expected result that capping agents reduce crystal growth, in our sonochemical synthesis, ETG and CA slightly increased the ZnO crystal sizes to 22 and 20 nm, respectively. This suggests that ETG and CA may form complexes with zinc ions or alter the local environment during synthesis, thereby promoting larger crystal formation. In contrast, the use of the surfactants PVP or OA, did not significantly affect the crystal size, 17 and 18 nm, respectively, similar to the 17 nm observed in the Bare-ZnO sample. In terms of the ZnO cell parameters, there were no significant changes, and the cell volume remained consistent among all the synthesized samples, with a value of 48 Å^3^.

Micrographs depicting the morphologies of the synthesized samples, both with and without organic modifiers, are displayed in Figure 2. Bare-ZnO, ZnO-OA, and ZnO-PVP exhibit a plate-like structure. Specifically, bare-ZnO (Figure 2(a)) shows stacked plates, while ZnO-OA (Figure 2(d)) displays a flower-like structure. ZnO-PVP (Figure 2(e)) reveals small particles dispersed over the plate-like structures. In contrast, the morphology of ZnO-CA (Figure 2(b)) undergoes a significant transformation, since agglomerated particles appear over the plate-like structure which is already almost unnoticed. Conversely, the ZnO-ETG (Figure 2(c)) presented a completely different morphology, consisting of oval-shaped particles with sizes around 100 nm. Therefore, ethylene glycol appears to inhibit the formation of the plate-like structure of ZnO, favoring the development of smaller oval-shaped particles. Additionally, the morphology observed in the commercial ZnO exhibits an almost spherical shape, as depicted in Figure S2 of the Supporting Information (SI), with a polydisperse distribution, indicated by the presence of different particle sizes.

**Figure 2:**
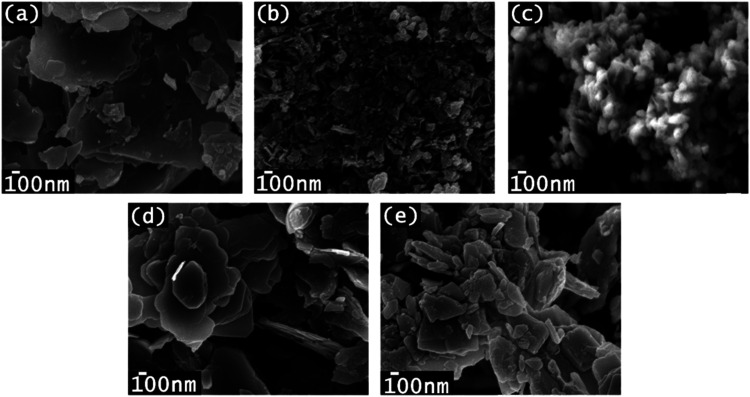
SEM micrographs taken at 50,000× magnification and a voltage of 5 V of the ZnO synthesized without and with capping agents: (a) Bare-ZnO, (b) ZnO-CA, (c) ZnO-ETG, (d) ZnO-OA, and (e) ZnO-PVP.

The change in morphology when using modifying agents can be attributed to the different functional groups present in each molecule. These functional groups provide various active sites during the nucleation and growth process of the ZnO, leading to interactions between the hydrophilic groups and the surface hydroxyl groups through dipole–dipole interactions, hydrogen bonding or Van Der Waals interactions.^38^ For instance, the use of citric acid inhibits the formation of plate-like structures and promotes the formation of smaller particles by utilizing its four coordination sites.^39,40^ ZnO-PVP exhibits a morphology similar to the bare-ZnO; the presence of PVP molecular chains hinders the growth of large plate-like structures, resulting in the formation of smaller particles and a slower crystal growth. In the case of oleic acid, clusters formed during the reaction tend to aggregate, resulting in a decrease in their surface area.^41^ This aggregation process leads to the formation of microparticles, which in turn act as sites for the formation of microflowers.^42–44^ Conversely, when ethylene glycol is utilized, the presence of this compound minimizes ionic diffusion. As a result, the structural formation occurs at a slower rate, inhibiting two-dimensional growth and consequently leading to the formation of oval-shaped structures.

TEM micrographs presented in Figure 3 also show distinct morphologies depending on the organic additive used in the synthesis. In the upper row of the figure, ZnO-CA and ZnO-ETG exhibit more spherical to oval-shaped particles, whereas Bare-ZnO, ZnO-OA, and ZnO-PVP show more elongated, rod-like morphologies. Based on TEM images, approximate particle sizes were estimated. ZnO-OA appeared to have the smallest average size (around 25 nm), while ZnO-PVP seemed to exhibit the largest (approximately 70 nm). Bare-ZnO (≈40 nm), ZnO-CA and ZnO-ETG (≈50–60 nm) showed intermediate sizes, with ZnO-CA and ZnO-ETG possibly having broader size distributions. The polycrystalline nature of all the samples is more evident in the higher-resolution images shown in the lower part of Figure 3, where well-defined lattice fringes indicate the presence of crystalline domains.

**Figure 3:**
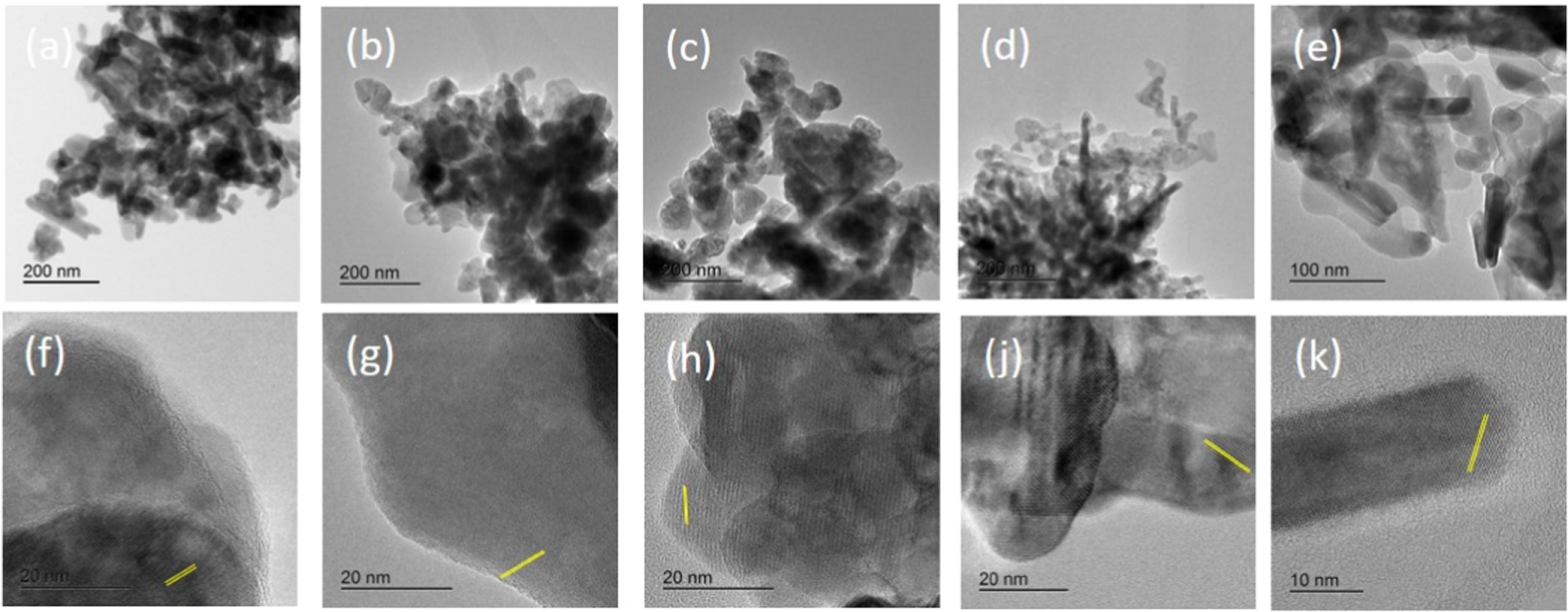
TEM micrographs of ZnO nanoparticles synthesized with different capping agents: (a, f) Bare-ZnO, (b, g) ZnO-CA, (c, h) ZnO-ETG, (d, j) ZnO-OA, and (e, k) ZnO-PVP. Top row: Low-magnification images. Scale bars: 200 nm for (a–d), 100 nm for (e). Bottom row: High-resolution TEM images. Scale bars: 20 nm for (f–j), 10 nm for (k).

Additionally, the EDS spectra (Figure S4 in the SI) show that Zn and O are the only elements detected in all samples. Any traces of organic molecules, if present, may not have been detected due to the low atomic number of their constituent elements.

### Hydrodynamic Diameter and ζ-Potential

To determine the hydrodynamic size, the synthesized ZnO materials with and without capping agents were dispersed according to the previously outlined dispersion protocol. In all cases, numerous aggregates with large particle sizes were observed, as shown in Figure 4 (left side). Notably, ZnO synthesized with ethylene glycol exhibited the smallest hydrodynamic size, measuring (369 ± 18) nm. Conversely, ZnO-PVP presented a larger hydrodynamic particle diameter of (620 ± 50) nm.

**Figure 4:**
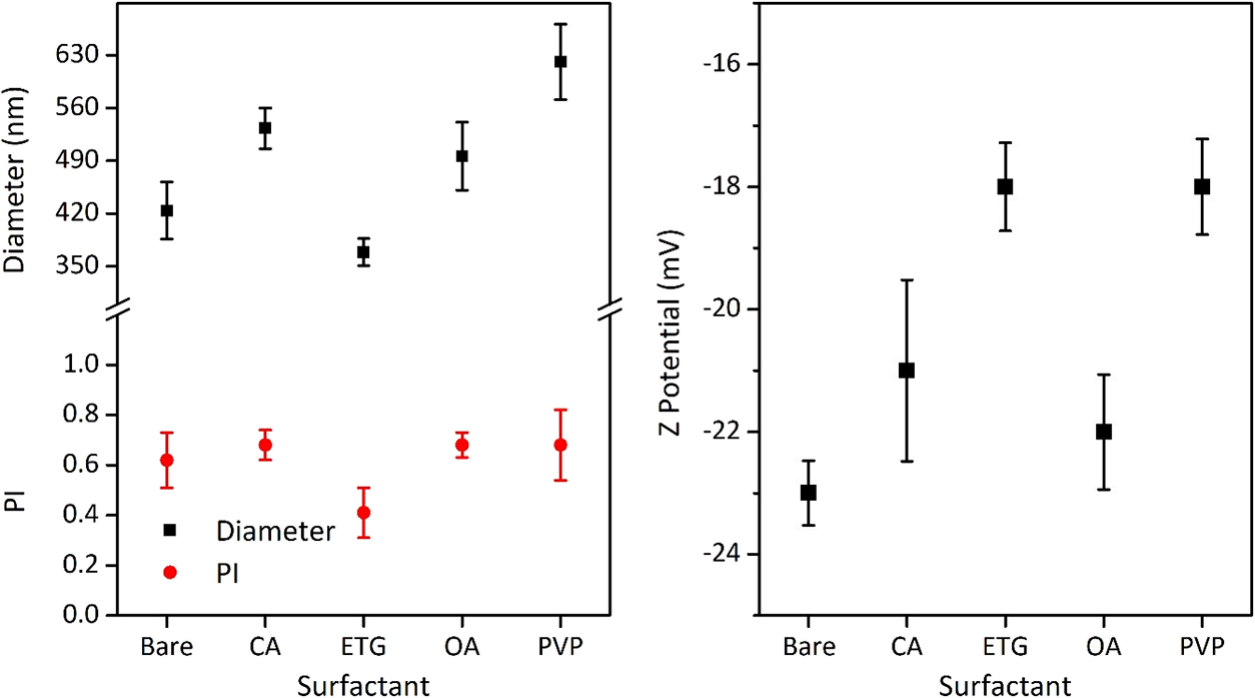
Left side: Hydrodynamic diameter and polydispersity index (PI). Right side: ζ-potential for ZnO particles synthesized with and without capping agents and dispersed in water at a neutral pH.

Additionally, all the samples presented a wide range of aggregates sizes, as indicated by polydispersity index values exceeding 0.4. For comparative analysis, measurements of the hydrodynamic diameter were conducted on commercially available ZnO particles. The particle size of our nanoparticles was found to be larger compared to the commercial ZnO, which measured (187 ± 9) nm. This disparity could be attributed to the variations in the synthesis protocol employed.

As previously mentioned, the determination of surface charge is crucial for understanding the colloidal stability of the synthesized ZnO samples. Electrophoretic mobility measurements were conducted at pH 7, as depicted in Figure 4 (right side). It was observed that all the samples exhibited negative ζ-potentials ranging between −18 and −23 mV due to the presence of negatively charged groups on the surface of the ZnO, such as OH^–^ groups generated by the excess of NaOH used during the synthesis process.^45–47^ As can be observed, the variation in ζ-potential among the nanoparticles synthesized with different organic molecules is not substantial. The lowest absolute values, around −18 mV, were observed for nanoparticles synthesized with neutral molecules such as PVP and ETG. In contrast, the highest ζ-potentials, around −23 mV, were recorded for bare ZnO nanoparticles and those functionalized with anionic molecules like OA and CA. In contrast, commercial ZnO particles displayed a positive ζ-potential of (20 ± 1) mV, indicating the presence of positively charged surface groups due to the adsorption of H^+^ groups for pH values smaller than the isoelectric point, that is around 9–10.^48^

### Optical Properties

Based on diffuse reflectance ultraviolet–visible spectroscopy (DRUV–vis) measurements, it was determined that all samples exhibit an absorption edge at approximately 368 nm (Figure 5, left-side). This characteristic absorption edge corresponds to ZnO with a hexagonal wurtzite structure and is associated with direct allowed electronic transitions between oxygen and zinc (O_2p_ → Zn_3d_).^49,50^ However, an intriguing continuum absorption band in the visible region (410–600 nm) was observed specifically in the ZnO-ETG and ZnO-OA samples. This phenomenon can be attributed to defects in the crystal structure, such as oxygen vacancies, zinc interstitials, and substitutional impurities. These defects introduce new energy levels within the band gap of ZnO, which act as ‘intermediate steps’ for the electrons. This allows the electrons to absorb photons with lower energy, become excited, and interact with molecular species to generate highly reactive intermediates, such as reactive oxygen species, hydroxyl radicals, and superoxide anions,^51–54^ thereby suggesting their potential for enhanced photocatalytic activity. In contrast, ZnO-CA and ZnO-PVP exhibit behavior similar to ZnO-Bare, with slight variations in absorption intensity, indicating that modification with these molecules does not significantly affect optical absorption in the visible region.

**Figure 5:**
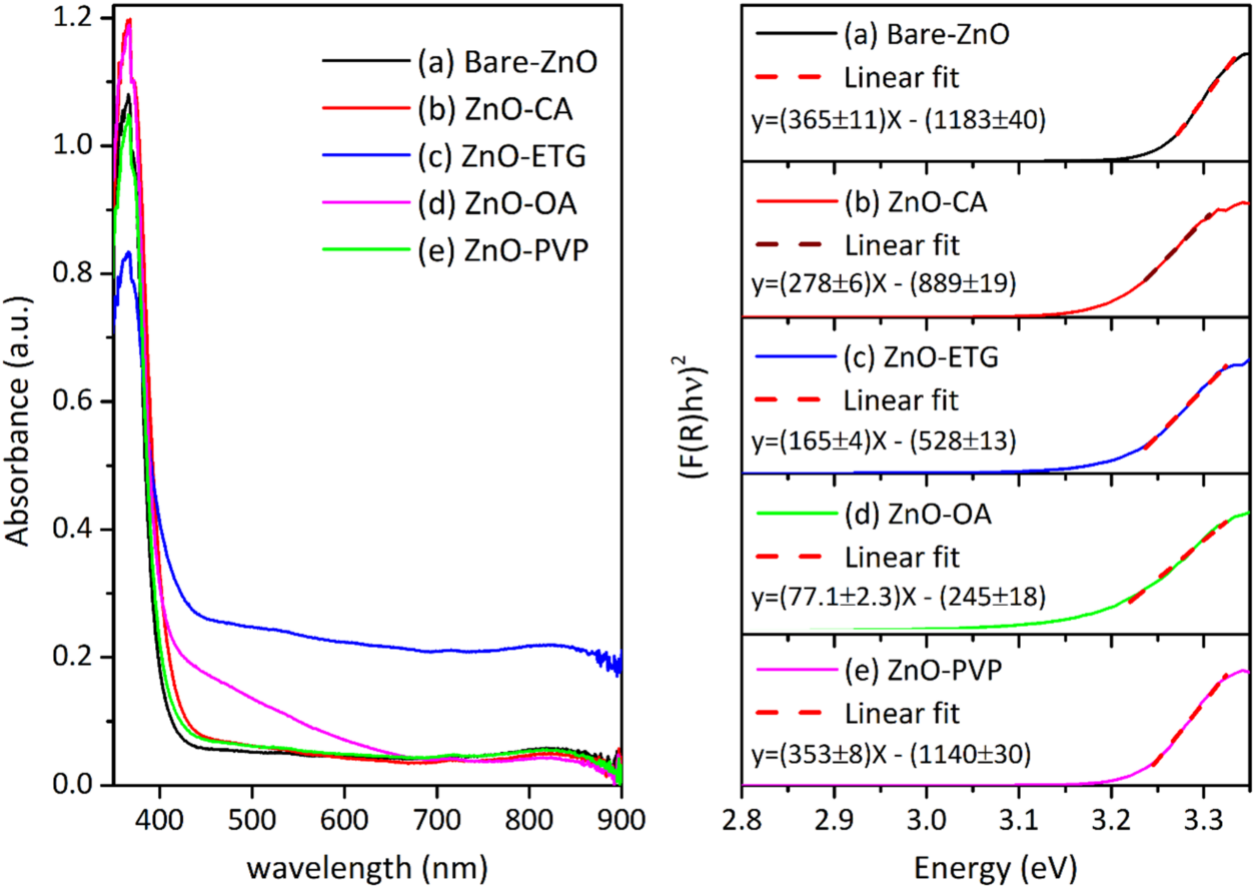
Left side: optical absorbance as a function of wavelength. Right side: (*F*(*R*)*h*υ)^2^ vs hυ for the ZnO samples synthesized without and with capping agent. The samples included in the plots are (a) Bare-ZnO, (b) ZnO-CA, (c) ZnO-ETG, (d) ZnO-OA and (e) ZnO-PVP.

The optical band gap was determined by estimating the intercept of the tangents to the plots (*F*(*R*)*h*υ)^2^ vs *h*υ (Figure 5, right-side) when (*F*(*R*)*h*υ)^2^ = 0. Additionally, the Urbach energy (*E*_u_) was obtained by calculating the reciprocal of the slope in the ln­(*F*(*R*)) versus *h*υ plot, where *F*(*R*) represents the reflectance, as shown in Figure 6. The choice of organic modifier not only influenced the morphology and crystal size but also affected the band gap and Urbach energy of ZnO. The use of ETG and PVP resulted in a slight decrease in the band gap of ZnO to 3.21 and 3.23 eV, respectively, while CA and OA induced a more pronounced decrease to 3.19 and 3.18 eV, respectively, compared to the bare-ZnO, 3.25 eV. The observed narrowing may be attributed to increased defect density or surface states introduced during the sonochemical synthesis. The stronger red shift in CA- and OA-modified ZnO suggests a more pronounced alteration of the electronic structure, likely due to their stronger coordination with Zn^2+^ and potential promotion of oxygen vacancy formation. The commercial ZnO shows the lowest band gap, 3.16 eV, as shown in Figure S3 of the SI. The difference in band gap between the samples obtained here and the commercial ZnO could be attributed to the presence of several defects in the synthesized samples.^55^

**Figure 6:**
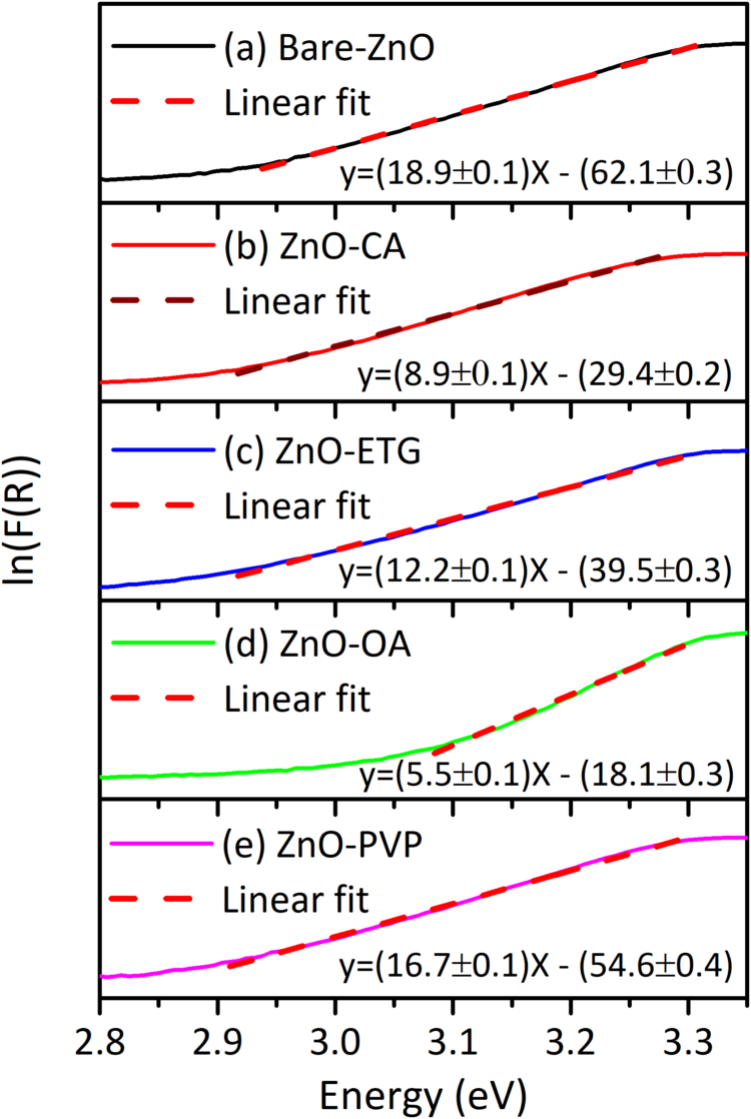
Urbach energy of the ZnO synthesized without and with capping agents; (a) Bare-ZnO, (b) ZnO-CA, (c) ZnO-ETG, (d) ZnO-OA and (e) ZnO-PVP.

Although organic modifiers have small impact on the band gap (from 3.18 to 3.25 eV), they do influence the formation of defects, which was confirmed through the calculation of Urbach energy and photoluminescence analysis (see Figure S5 of the SI). Citric acid, for instance, can coordinate with Zn^2+^ ions, leading to the formation of oxygen vacancies via Zn-complexation. This results in an increase in Urbach energy from 53 to 82 meV, indicating the presence of more disordered states. ETG, a polar molecule, can also promote zinc interstitials and oxygen vacancies due to its influence on crystal growth,^56,57^ increasing Urbach energy to 112 meV. Similarly, oleic acid, a hydrophobic surfactant, can hinder uniform crystal growth, promoting the creation of oxygen vacancies and zinc interstitials.^58^ This results in an even higher Urbach energy of 183 meV. In contrast, PVP reduces defects by stabilizing the ZnO structure and preventing agglomeration, thus minimizing the formation of zinc interstitials and oxygen vacancies,^59^ which leads to a similar Urbach energy (60 meV) to the bare ZnO.

Therefore, using capping agents during the synthesis of ZnO not only modifies the morphology and size of the particles but also alters their optical and microstructural properties.

### Photocatalytic Activity

The photocatalytic performance of the samples was first evaluated by measuring the degradation of reactive black 5 under natural pH conditions, with results shown in Figure 7. Remarkably, ZnO-ETG exhibited the highest photoactivity, achieving complete degradation within just 40 min. In contrast, ZnO-OA, bare ZnO, ZnO-CA, and ZnO-PVP achieved degradation percentages of 97, 95, 93 and 93%, respectively, after two hours of reaction, as outlined in Table 2. Notably, both bare ZnO and ZnO-CA samples exhibited almost identical degradation percentages within the same time frame. Additionally, using commercial ZnO as the photocatalyst resulted in only 43% degradation under the same conditions. Thus, under these degradation conditions, the synthesized samples demonstrated superior photocatalytic activity compared to commercial ZnO. An intriguing result is that the activity of ZnO appears to depend not only on its optical properties, defects, and sizes, but also on morphology. For example, despite ZnO-ETG and ZnO-OA having similar optical properties and hydrodynamic diameters (size in solution), ZnO-ETG forms oval-shaped particles whereas ZnO-OA forms microflowers. This variation suggests that ZnO activity also hinges on the interactions established between the Zn precursor and the organic additive during the synthesis reaction, which modify both the morphology of ZnO and the nature of additive interactions with pollutant molecules during the degradation process. In fact, materials synthesized with different modifying agents exhibit varying adsorption capacities after the system was kept in the dark for 30 min to reach equilibrium, as shown in the figure legend. Interestingly, there appears to be a correlation between adsorption and degradation, the higher the adsorption, the greater the photocatalytic performance. Additionally, we measured the maximum adsorption capacity (*Q*_max_) of our materials under these conditions (see Figure S6 in the Supporting Information), and observed a correlation between *Q*_max_ and the degree of adsorption.

**Figure 7:**
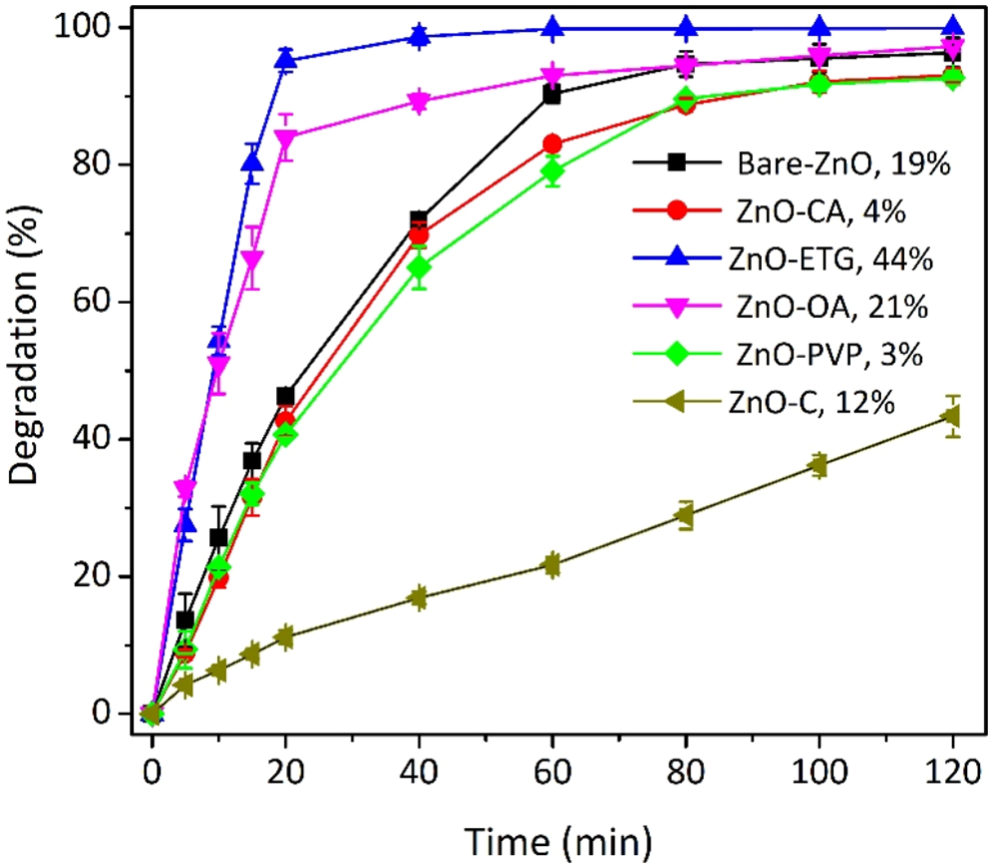
Degradation percentages of the RB5 azo dye at natural pH (6.8) employing as photocatalyst: Bare ZnO, ZnO-CA, ZnO-ETG, ZnO-OA, ZnO-PVP, and commercial ZnO. The dye adsorption percentage on each catalyst after 30 min in darkness, prior to irradiation, is also indicated alongside the legend.

**Table 2:** Optical Properties and Photocatalytic Performance in Degrading RB5 Azo Dye under Natural, Acidic, and Basic pH Conditions after Two Hours of Reaction of ZnO Nanoparticles Synthesized without and with Capping Agents

	optical properties		% degradation			% mineralization		
sample	*E*_g_ (eV)	Urbach energy (meV)	pH 6.8	pH 3	pH 10	pH 6.8	pH 3	pH 10
bare ZnO	3.25	53	95	20	100	78	27	92
ZnO-CA	3.19	82	93	18	90	87	20	78
ZnO-ETG	3.21	112	100	31	100	95	35	97
ZnO-OA	3.18	183	97	24	100	90	25	95
ZnO-PVP	3.23	60	93	12	100	76	18	81
commercial	3.16	179	43	93	58	-	-	-

The pH of a solution is well-known to play a crucial role in photocatalytic processes, affecting the interaction between the surface charges of the photocatalyst and dye molecules. Therefore, we investigated the impact of pH on the photocatalytic activity of ZnO synthesized both with and without capping agents. To this end, the degradation of the same dye was also studied at acidic (pH 3) and basic (pH 10) conditions. These pH values were achieved by adding 0.1 M H_2_SO_4_ and 0.1 M NaOH, respectively, to the reaction medium before initiating the adsorption–desorption process between the dye and the photocatalyst.

The results for degradation under these pH conditions are depicted in Figure 8. Comparison with results obtained at the standard pH of the dye (pH 6.8) revealed that employing an acidic medium reduced the photocatalytic activity of all synthesized samples. Among them, the sample synthesized with oleic acid and ethylene glycol demonstrated the highest degradation percentage, around 25–30%, within a 120 min time frame. Conversely, the sample synthesized with PVP exhibited the lowest photocatalytic activity, achieving a degradation percentage of only 12% approximately. It is worth noting that commercial ZnO exhibited an increased degradation of the dye (93%) under these acidic conditions, as illustrated on the upper part of Figure 8.

**Figure 8:**
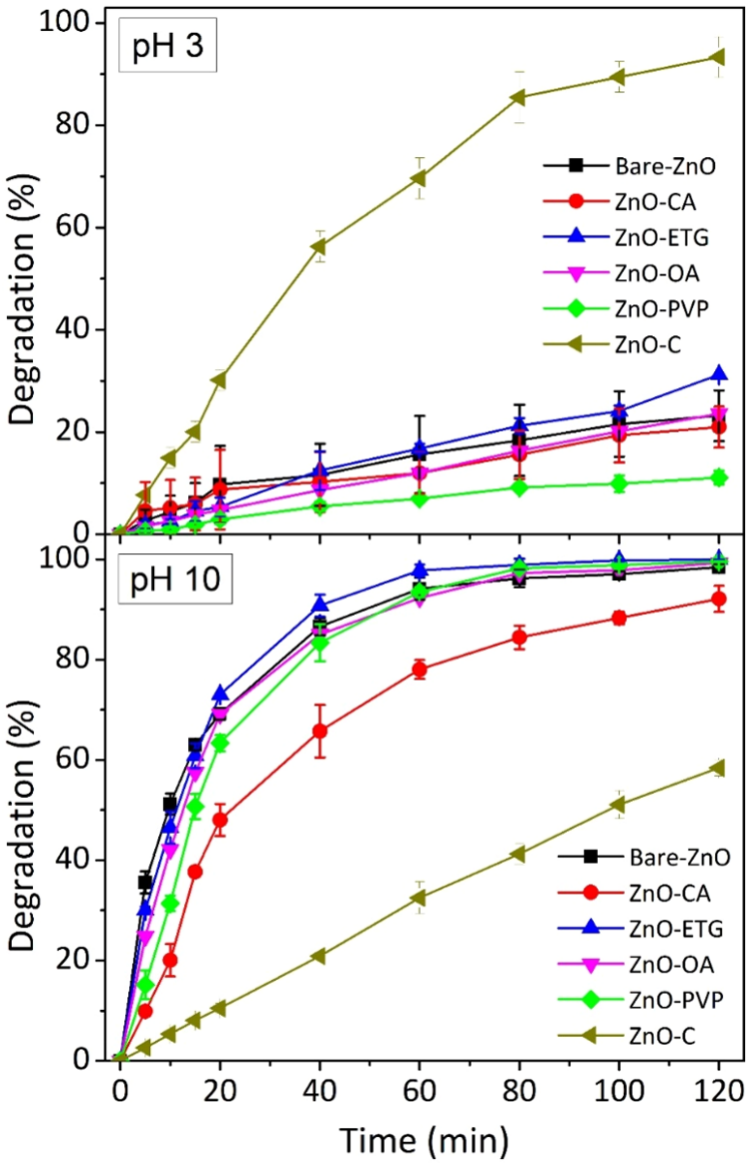
Degradation percentages of the RB5 azo dye at pH 3 and at pH 10 using the following photocatalysts: Bare ZnO, ZnO-CA, ZnO-ETG, ZnO-OA, ZnO-PVP, and commercial ZnO.

Conversely, when a basic medium was employed, a behavior similar to the degradation reactions conducted at the standard pH was observed (Figure 8, lower part). Nearly all the samples exhibited complete degradation of the dye, except for ZnO-CA, which showed slightly lower degradation compared to the reactions at the standard pH. Additionally, commercial ZnO displayed a degradation percentage of 58%, slightly higher than that observed at the standard pH.

The decrease in photocatalytic activity of the synthesized samples in an acidic medium can be attributed to the dissociation of ZnO into Zn^2+^ and O^2–^ ions.^11,16,60–62^ This dissociation leads to the presence of ions in the solution, resulting in a change to a homogeneous reaction mechanism. Consequently, there is an inhibition in the formation of hydroxyl radicals, which are fundamental for the degradation reactions. On the other hand, a basic pH promotes the presence of more hydroxyl ions. These ions, when reacting with the holes, generate hydroxyl radicals that enhance the photodegradation of RB5.^20,63,64^ However, when the pH is close to neutral, the photocatalytic efficiency is attributed to the presence of hydroxyl groups on the ZnO surface. Furthermore, the observed behavior of commercial ZnO, which is opposite to that of the synthesized materials, can be attributed to the difference in surface charge between the synthesized ZnO materials, which have a negative surface charge in contrast to the positive surface charge of the commercial ZnO. The mineralization results, obtained from total organic carbon (TOC) measurements (see Table 2), show a reasonable agreement with the degradation efficiencies, as it is shown in Figure S7 of the Supporting Information.

Hence, pH variation significantly influences the degradation process of both synthesized ZnO and commercial ZnO, as it affects the electrostatic forces between the ZnO surface groups and dye molecules.^38,43,65,66^ To gain further insight into the relationship between pH and the photocatalytic activity of ZnO, ζ-potential measurements were carried out in acidic and basic media. As depicted in Table 3, under acidic conditions, the synthesized samples display a ζ-potential close to the isoelectric point, indicating proximity to the zero-charge point, and the system become unstable decreasing the dispersion degree of all the synthesized materials as indicated by measurements of their hydrodynamic size. Conversely, at pH 10, the samples present a higher ζ-potential, maintaining their negative charge and hydrodynamic size obtained at natural pH.

**Table 3:** ζ-Potential (mV) of Synthesized ZnO with and without Capping Agent at Various pH

sample	pH 3	pH 7	pH 10
bare ZnO	8.1 ± 0.4	–23.0 ± 0.5	–56.3 ± 4.6
ZnO-CA	7.8 ± 1.0	–20.8 ± 1.5	–56.3 ± 3.1
ZnO-ETG	2.6 ± 0.5	–18.1 ± 0.7	–56.5 ± 1.8
ZnO-OA	5.8 ± 0.8	–21.9 ± 0.9	–47.6 ± 0.5
ZnO-PVP	2.6 ± 0.5	–18.1 ± 0.7	–56.5 ± 1.8
commercial	31.0 ± 2.6	20.2 ± 1.0	2.1 ± 0.7

The negative surface charge of the samples limits the absorption of the anionic pollutant molecules onto the ZnO surface, preventing full occupancy of the active sites.^67^ However, under acidic conditions, RB5 molecules can strongly adhere to the ZnO surface, inhibiting the formation of hydroxyl radicals and thereby reducing dye degradation.^68^ Conversely, commercial ZnO, with its positive surface charge, exhibits behavior opposite to that of the synthesized samples. Nevertheless, regardless of pH, ZnO-ETG consistently showed the highest degradation rates across all the pH studies among the synthesized samples.

Returning to the point addressed in the introduction, another significant factor influencing the photocatalytic activity of ZnO is the nature of the pollutant. As part of a comparative study, we evaluated the photocatalytic efficiency of our materials in degrading methylene blue at a concentration of 20 ppm, a reaction widely studied and documented in specialized literature. In contrast to RB5, this dye is cationic, highly stable, and extensively used in pharmaceutical and textile industries.^69,70^ The degradation results of methylene blue at natural pH (pH = 7.4) are presented in Figure 9, along with the dye adsorption capacity of each photocatalyst, measured after 30 min of stirring in the dark, which is indicated next to its label. As illustrated, the addition of organic modifiers in the synthesis enhanced the photocatalytic activity of the materials. ZnO synthesized without a capping agent exhibited the lowest performance, with a degradation efficiency of 87%. In contrast, ZnO-ETG and ZnO-PVP displayed the highest efficiency, achieving complete degradation of MB within 100 min. ZnO-CA and ZnO-OA reached a degradation percentage of 96%, comparable to that observed for the commercial ZnO.

**Figure 9:**
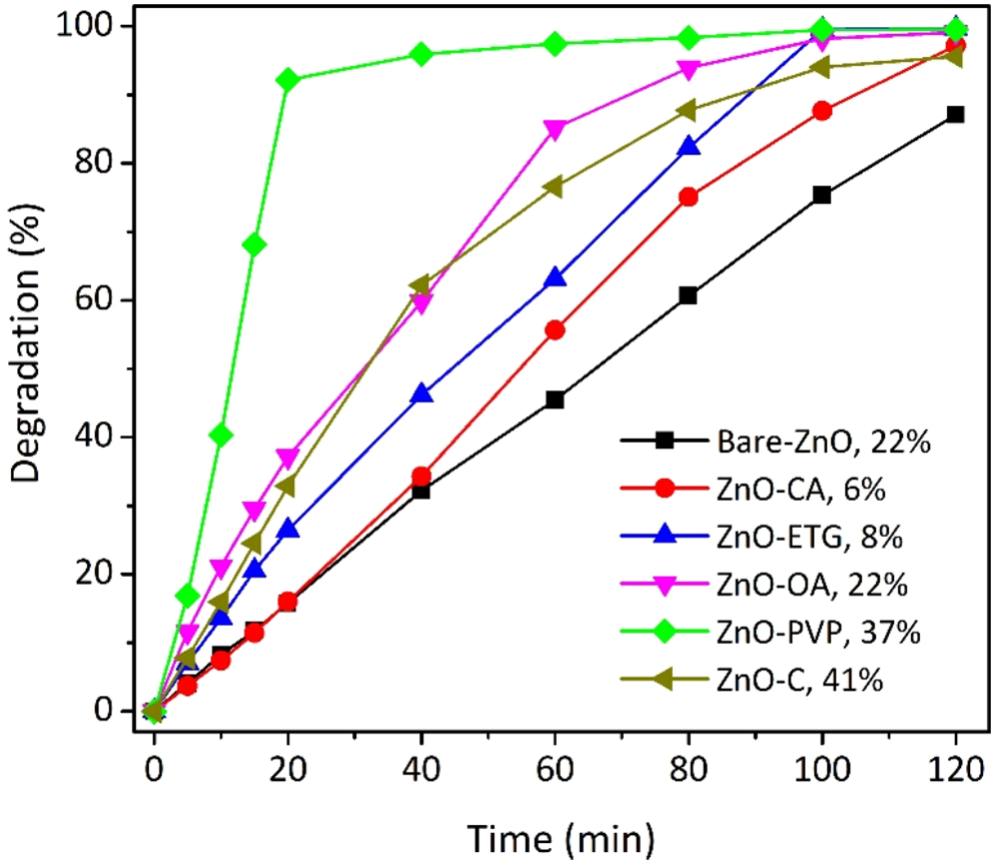
Degradation of the MB dye at natural pH (7.4) employing as photocatalyst (a) Bare ZnO, (b) ZnO-CA, (c) ZnO-ETG, (d) ZnO-OA, (e) ZnO-PVP, and (f) Commercial ZnO. The dye adsorption percentage on each catalyst after 30 min in darkness, prior to irradiation, is also indicated alongside the legend.

As observed, the functionalization of ZnO with different agents modifies both the adsorption and photodegradation of methylene blue (MB). However, in contrast to RB5 degradation, MB degradation does not appear to depend solely on its adsorption onto the photocatalyst surface, but also on other key factors, such as the generation of highly reactive species (hydroxyl radicals ^•^OH and superoxide anions ^•^O_2_^–^) and the efficiency of charge transfer.^71^ This is evident from the fact that higher dye adsorption does not necessarily result in greater degradation. For instance, while the ZnO-PVP sample shows both the highest MB adsorption and the greatest photocatalytic efficiency, the ZnO-ETG sample, despite its low adsorption capacity, is still capable of fully degrading the dye.

The variation in photocatalytic activity observed between ZnO synthesized with and without capping agent in the degradation of RB5 and MB can primarily be attributed to differences in the chemical structures of the dyes, as illustrated in Figure S8 (SI), as well as the optical and surface characteristics of the synthesized materials. RB5 contains two azo groups (−N=N−) with multiple aromatic rings and sulfonate groups, whereas MB has an aromatic structure comprising three benzene rings linked together by nitrogen and sulfur atoms, with the central nitrogen atom forming part of a heterocyclic ring.^72,73^ Additionally, the reaction medium also plays a significant role, as these properties collectively contribute to different reaction pathways.^74^

Despite this, all the samples presented good photocatalytic activity for degrading both dyes at natural pH. In particular, ZnO-ETG had a good photocatalytic behavior with both contaminants. The superior photocatalytic activity of ZnO-ETG can be attributed to its different morphology. Unlike the others, ZnO-ETG exhibited an oval morphology, while the others predominantly displayed lamellar morphologies. Furthermore, despite having the largest crystal size, ZnO-ETG had the smallest hydrodynamic diameter (369 ± 18) nm, resulting in a larger contact area due to the decreased size of the dispersed material. In addition, ZnO-ETG presents optical absorbance in the visible region of the electromagnetic spectrum and possesses the highest Urbach energy. The higher Urbach energy suggests the presence of a larger number of defects in the band gap states, along with a greater abundance of hydroxyl groups, as seen in its infrared spectrum illustrated in Figure S9 from the Supporting Information. These hydroxyl groups are crucial for initiating oxidation–reduction reactions, which play a pivotal role in the degradation of organic pollutants. Their presence is essential for promoting the generation of highly reactive species necessary for the degradation process. Furthermore, Raman spectroscopy (see Figure S10 in the Supporting Information) supports this superior performance, since ZnO-ETG shows a greater presence of structural defects (evidenced by the band at 560 cm^–1^) and surface modifications (indicated by the broad band in the 1040–1140 cm^–1^ region).

As previously mentioned, the hydrodynamic size is a critical factor influencing the photocatalytic activity of ZnO and is strongly affected by its morphology. Oval-shaped particles benefit from better dispersion and higher surface area, while lamellar structures tend to agglomerate, resulting in a larger hydrodynamic size. As shown in Figure 10(a), smaller hydrodynamic sizes increase the degradation percentage of RB5 azo dye, primarily due to the increased surface area and more active sites. Therefore, achieving smaller sizes without aggregation is crucial for higher degradation of organic pollutants. Hence, at pH 3, where the nanoparticles are aggregated and exhibit a very high hydrodynamic radius, the degradation percentage is significantly reduced. ζ-Potential is another important factor related to the dispersion degree and stability of ZnO, and consequently, its photoactivity, as seen in Figure 10(b). Samples with low ζ-potential values are colloidally unstable and display large aggregates, as confirmed by the high hydrodynamic size values. Thus, managing ζ-potential through pH adjustment and surface modification can optimize the degradation efficiency of RB5. Additionally, Urbach energy significantly impacts the photocatalytic activity of ZnO, as shown in Figure 10(c). Urbach energy indicates the degree of disorder in semiconductors, where higher values generally correlate with enhanced light absorption, leading to improved degradation of RB5 azo dye. However, excessive disorder can decrease ZnO’s photoactivity due to the introduction of numerous defects, such as vacancies, interstitials, and antisites. These defects can create localized states within the band gap or act as recombination centers for the photogenerated electron–hole pairs, preventing these charge carriers from contributing to the photodegradation process. Moreover, RB5 dye degradation was also tested under solar irradiation using the material with the best photocatalytic performance, ZnO-ETG. After 4 h, the dye degradation reached 71%. This promising result suggests that ZnO-ETG could be effectively used for wastewater treatment in the textile industry.

**Figure 10:**
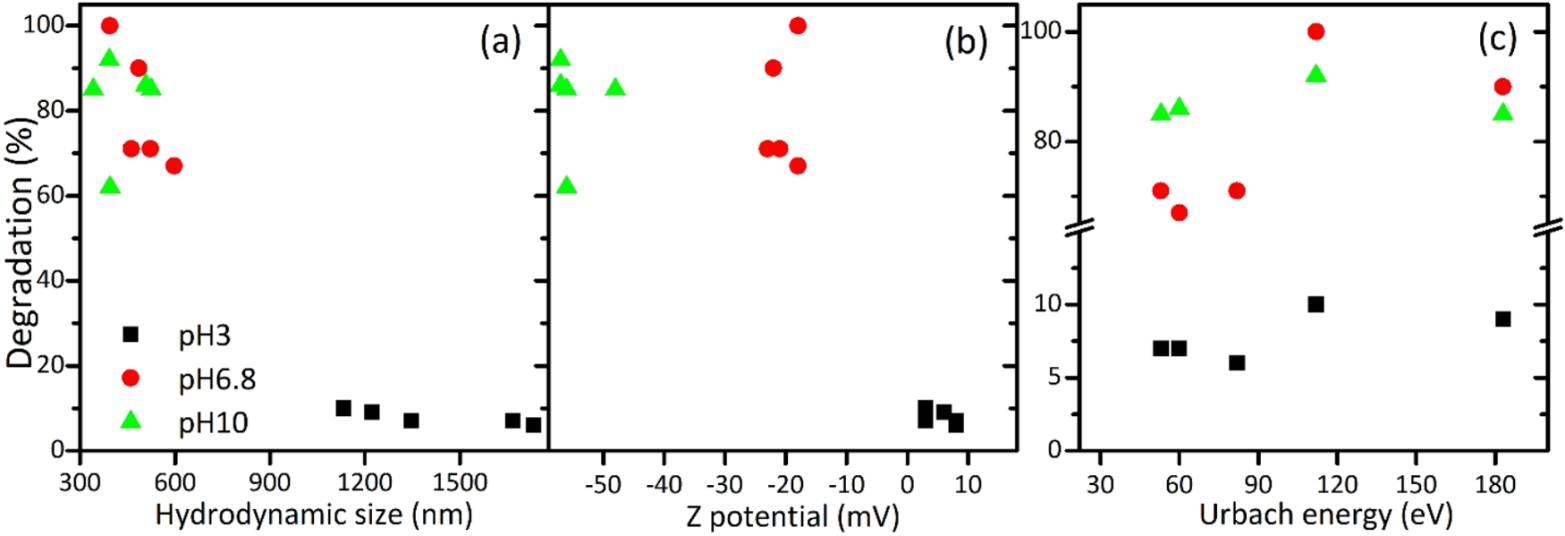
Relationship between the percentage of degradation of RB-5 dye measured at pH 3 (black squares), pH 6.8 (red circles), and pH 10 (green triangles) with (a) hydrodynamic size, (b) ζ-potential, and (c) Urbach energy for all synthesized ZnO materials.

To enable a direct comparison of the photocatalytic efficiency among the synthesized materials and with values reported in the literature for other catalysts, the kinetic rate constants for the degradation of MB and RB5 were determined, data shown in Table 4. These constants were obtained by fitting the experimental data to a pseudo-first-order kinetic model, which is commonly used in dye degradation studies.^75^ The kinetic constant (*k*_c_) was calculated from the slope of the linear plot of ln­(*C*_0_/*C*) versus time (*t*), where *C*_0_ is the initial dye concentration and *C* is the concentration at a given time.

**Table 4:** Comparative Photocatalytic Performance of Different Materials for the Degradation of Methylene Blue (MB) and Reactive Black 5 (RB5)

catalyst	catalyst concentration (g/L)	dye and concentration (ppm)	kinetic constant (min^–1^)	degradation % and time	refs
ZnO	0.1	MB at 10 ppm	0.0027	99.5%, 50 min	^19^
TiO_2_	0.15	MB at 8 ppm	0.0047	62%, 220 min	^76^
rGO-Fe_3_O_4_–TiO_2_		MB at 8 ppm	0.0187	99%, 55 min	
CeO_2_	1	MB at 15 ppm	---	77%, 120 min	^77^
CuO		MB at 15 ppm	---	15%, 120 min	
ZnO		MB at 15 ppm	---	98%, 120 min	
bare-ZnO	0.4	MB at 3 ppm	---	70%, 90 min	^22^
ZnO-CTAB			---	86.3%, 90 min	
ZnO-PEG			---	90.8%, min	
ZnO-AOT			---	82.8	
ZnO	4	MB at 10 ppm		70%, 85 min	^78^
CuO				50%, 85 min	
CuO/ZnO				97%, 85 min	
WO_3_/TiO_2_	0.25	RB5, 30 ppm	0.00225	73%, 60 min	^79^
Ag/ZnO	0.5	RB5, 10 ppm	0.0017	72%, 780 min	^80^
CuO–DMF	0.2	RB5, 10 ppm	0.0047	68.95%, 300 min	^81^
CuO-MeOH			0.0097	95.19%, 300 min	
CuO–DMSO			0.0052	77.31%, 300 min	
WO_3_	1	RB5, 5 ppm	0.0052	83%, 120 min	^82^
	0.75		0.0058	100%, 120 min	
	0.5		0.0057	91.44%, 120 min	
Ag_3_PO_4_	0.5	RB5, 50 ppm	---	27%, 120 min	^83^
bare-ZnO	1	RB5, 20 ppm	0.034	95%, 120 min	this work
		MB, 20 ppm	0.011	87%, 120 min	
ZnO-CA		RB5, 20 ppm	0.028	93%, 120 min	
		MB, 20 ppm	0.017	96% 120 min	
ZnO-ETG		RB5, 20 ppm	0.093	100%, 40 min	
		MB, 20 ppm	0.040	100%, 100 min	
ZnO-OA		RB5, 20 ppm	0.036	97%, 120 min	
		MB, 20 ppm	0.025	96%, 120 min	
ZnO-PVP		RB5, 20 ppm	0.029	93%, 120 min	
		MB, 20 ppm	0.045	100%, 100 min	

As we can see in Table 4, compared to other materials reported in the literature, the ZnO-based catalysts synthesized in this work exhibited competitive, and in some cases superior, photocatalytic performance. In the degradation of MB, the ZnO-ETG and ZnO-PVP samples achieved complete degradation (100%) within 100 min. In the case of RB5, the synthesized ZnO-ETG catalyst showed particularly remarkable performance, reaching 100% degradation in just 40 min with a high kinetic constant of 0.093 min^–1^. These results highlight the enhanced activity of the synthesized materials, especially for the degradation of RB5, where their efficiency surpasses, as far as we know, most previously reported photocatalysts.

## Conclusions

The selection of organic modifier (or absence thereof) during ZnO synthesis significantly influences its properties, particularly its morphology and optical properties. XRD results confirm the hexagonal wurtzite structure for all samples, with variations in crystal sizes and UV absorbance. Among the capping agents used as stabilizers, the use of ETG enhances the photocatalytic activity of ZnO in degrading RB5, followed by OA. This enhancement can be attributed to differences in morphology, particularly in the case of ZnO-ETG, which exhibits an oval particle morphology. The smaller particle size, coupled with a reduced hydrodynamic size, due to the use of this stabilizer, increases the surface area, rendering it an effective material for removing organic pollutants from aqueous solutions through processes like photocatalysis. The photocatalytic efficiency of the synthesized ZnO samples decreases in acidic media due to the dissociation of ZnO into Zn^2+^ and O^2–^ ions, leading to an inhibition in the formation of hydroxyl radicals and to an increase of the hydrodynamic size, which is related to the dispersion degree of the material due to the lowering of the ζ-potential, responsible of the electrostatic stability of the colloidal dispersion. Conversely, in basic media, there is a higher presence of hydroxyl ions, which facilitates a greater generation of radicals and a higher dispersion degree of the ZnO particles. These results contrast with the behavior of commercial ZnO, largely due to its distinct surface charge.

Hence, it was found that the morphology, optical properties, and presence of defects in ZnO play a crucial role in its interaction with RB5 azo dye and MB. The modifying agents used during synthesis could introduce functional groups on the ZnO surface, along with the generation of oxygen vacancies, altering the interactions between ZnO and different organic molecules. These factors significantly influence its photocatalytic activity in the degradation of dyes such as RB5 or MB.

Respect to the photocatalytic performance of our materials, ZnO-ETG and ZnO-PVP achieved complete degradation of MB within 100 min, and ZnO-ETG also showed an efficient performance in the removal of RB5, reaching full degradation in just 40 min. These results suggest that these materials could be considered as competitive alternatives to other photocatalysts reported in the literature, particularly for the treatment of RB5.

## Supplementary Material



## Data Availability

All the relevant data generated or analyzed during this study are included in this article.

## Notes

Diffraction pattern of commercial ZnO; SEM micrographs of commercial ZnO; Optical Absorbance of commercial-ZnO; EDS Spectra; Photoluminescence spectroscopy; RB5 adsorption Langmuir isotherms; RB5 mineralization degree; Chemical structure of RB5 and MB; Infrared spectra of ZnO NPs; Raman spectra (PDF)
