# Supplementary material for: Comparison of Photocatalytic Performance of Sonochemically Synthesized ZnO with Different Capping Agents
Source: ACS Omega. 2025 Jul 9;10(28):30181–93. doi: 10.1021/acsomega.5c00929 (PMC12290650; doi:10.1021/acsomega.5c00929)
Supplement: Supplementary file 1 [file ao5c00929_si_001.pdf]

Supporting Information

Comparison of photocatalytic performance of sonochemically synthesized ZnO with different capping agents

Tatiana Rodríguez-Flores, Isaías Hernández-Pérez, Gloria Elena de la Huerta-Hernández, Yadira Ayala-Parada, Jessica Guadalupe Cadena-Silva, and Catalina Haro-Pérez*

Departamento de Ciencias Básicas, Universidad Autónoma Metropolitana-Azcapotzalco, Av. San Pablo 420, C.P 02128, Ciudad de México, Mexico

*e-mail: cehp@azc.uam.mx

Commercial ZnO characterization

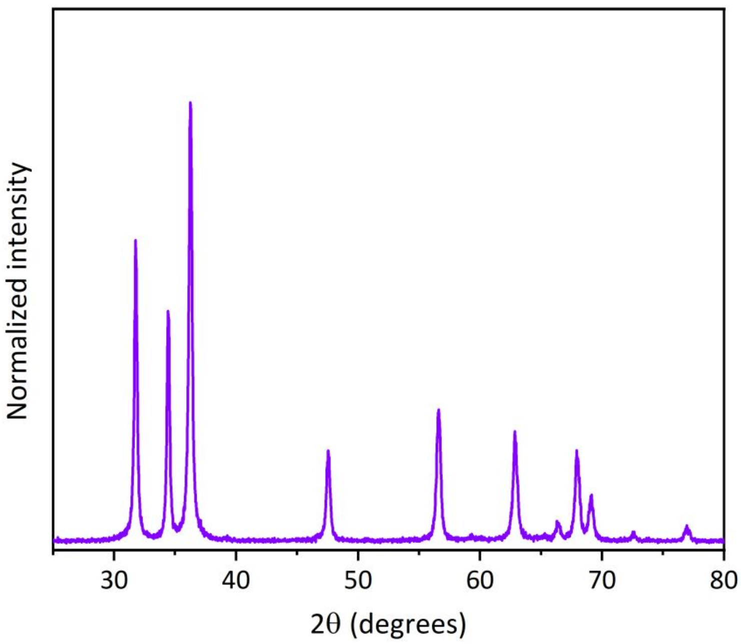

Fig. S1. Diffraction pattern of commercial ZnO

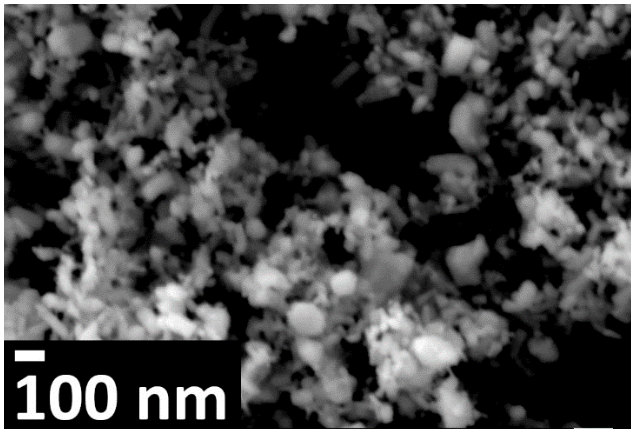

Fig. S2. Micrographs taken at 50,000 magnification and a voltage of 5V of the commercial ZnO.

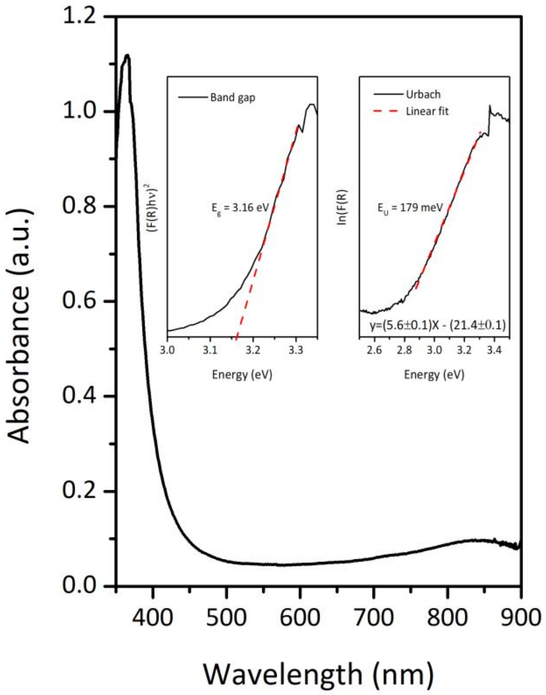

Fig. S3. Optical Absorbance of commercial-ZnO. Insets show the Band gap and Urbach energy.

EDS Spectra

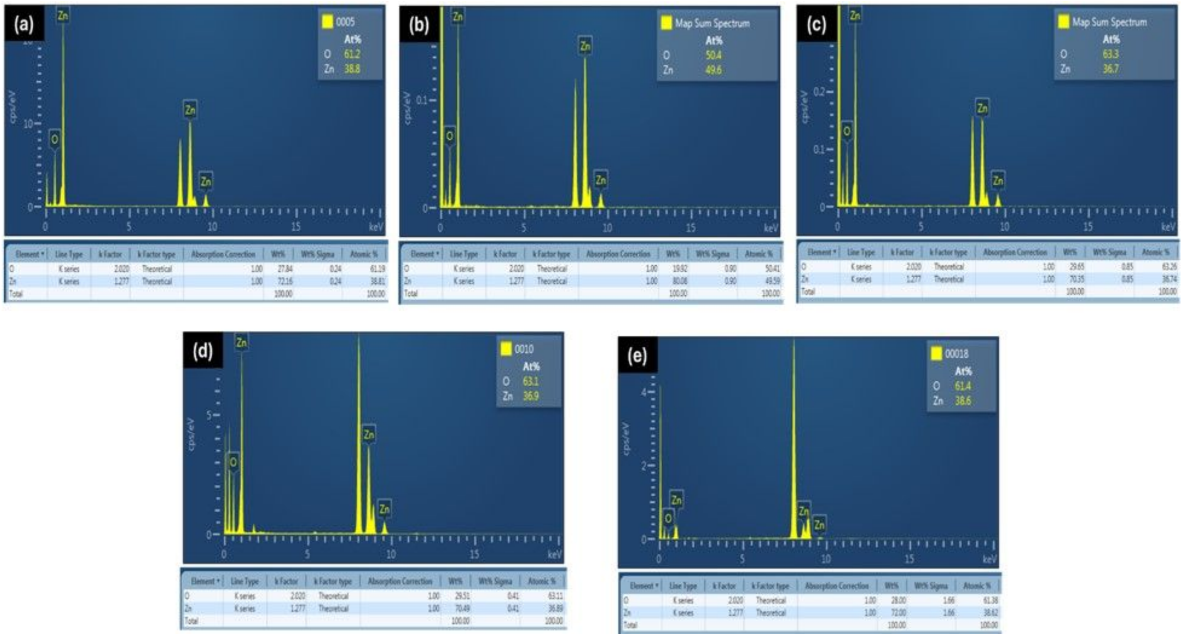

Fig. S4. EDS analysis of the ZnO synthesized without and with capping agent: (a) Bare-ZnO, (b) ZnO-CA, (c) ZnO-ETG, (d) ZnO-OA, and (e) ZnO-PVP

Photoluminescence spectroscopy

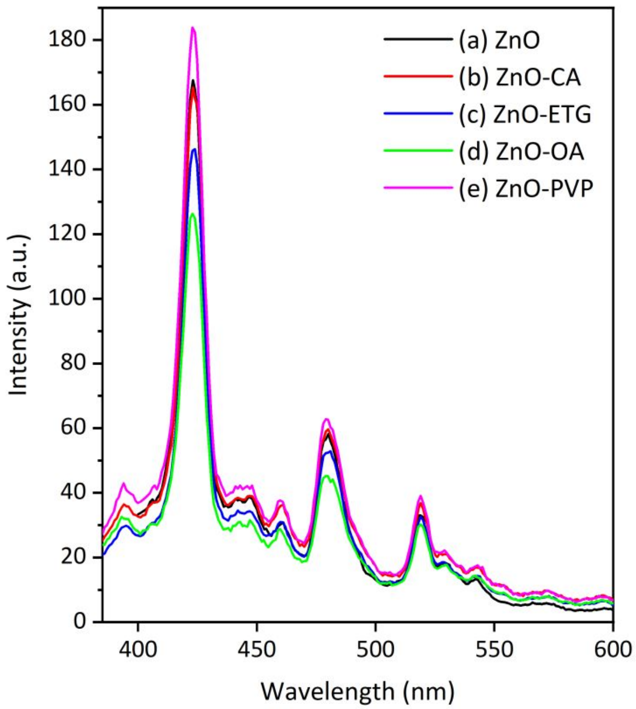

Fig. S5. Photoluminescence spectroscopy of the ZnO synthesized without and with capping agent; (a) Bare-ZnO, (b) ZnO-CA, (c) ZnO-ETG, (d) ZnO-OA, and (e) ZnO-PVP.

In the photoluminescence studies, performed at an excitation wavelength of 370 nm, it was found the presence of different types of defects such as interstitials and vacancies of zinc and/or oxygen. As can be seen in Fig. S4, the first band is located in the UV region (394 nm), and it is associated to the radiative recombination of e^- — h^+ pairs (photon emission), being the ZnO-PVP the sample with the highest intensity, while the lowest intensity is observed for the ZnO-ETG sample. On the other hand, the band at 423 nm is attributed to the transition of an electron from a donor level of zinc interstitial (Zn_i) to the upper level of the valence band. The emissions between 460-470 nm are attributed to the states of intrinsic defects such as zinc and oxygen vacancies, as well as Zn_i. Finally, the emission band located at 520 nm is primarily due to oxygen vacancies.

Adsorption kinetics of RB5 dye on the surface of the photocatalyst

The adsorption of Reactive Black 5 (RB5) onto the surface of ZnO nanoparticles (NPs) was studied using a batch-mode process in a glass reactor. A fixed photocatalyst concentration of 1.0 g/L was used with varying initial dye concentrations (10–100 ppm). The suspensions were stirred in complete darkness for 1 hour to reach adsorption equilibrium. After this period, the solutions were filtered through a 0.2 μm SFCA membrane filter, and the remaining dye concentration was measured at 597 nm using UV-Vis spectrophotometry.

The amount of dye adsorbed at equilibrium (q_e) was calculated using the following equation:

$$q_e = ((C_0 - C_e) / M) \times V \qquad (1)$$

where:
- C_0 and C_e (mg/L) are the initial and equilibrium dye concentrations,
- M is the mass of photocatalyst (g),
- V is the volume of the solution (L).

The Langmuir isotherm model was used to determine the maximum adsorption capacity ($Q_{\square ax}$), assuming monolayer adsorption on a homogeneous surface. Its linearized form is given by:

$$C_e / q_e = C_e / Q_{\square ax} + 1 / (K_L \times Q_{\square ax}) \quad (2)$$

From the plot of C_e/q_e versus C_e, the constants K_L (Langmuir constant) and $Q_{\square ax}$ (maximum adsorption capacity) were obtained by linear regression. Figure S5 shows the Langmuir isotherms for ZnO samples synthesized with and without modifying agent.

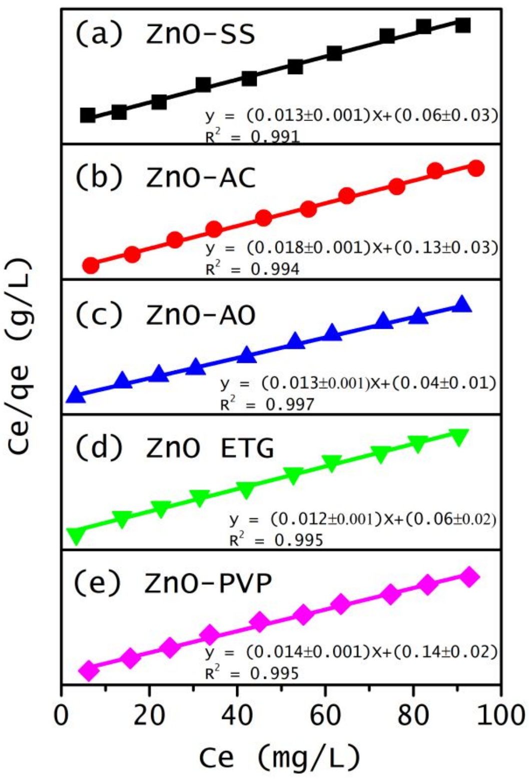

Fig. S6. Langmuir adsorption isotherms of ZnO synthesized with different capping agents for RB5: (a) ZnO-SS, no capping agent, (b) ZnO-CA, (c) ZnO-OA, (d) ZnO-ETG, and (e) ZnO-PVP.

The values of Qmax obtained are listed in the next table along the adsorption percentage found after 30 minutes of continuous stirring in the dark prior the degradation experiments.

Sample	% RB5 adsorption	RB5 Q_{max} (mg/g)	% RB5 degradation
Bare ZnO	19	76	96
CA-ZnO	4	54	93
ETG_ZnO	44	85	100
OA-ZnO	21	80	97
PVP-ZnO	3	69	92
Com-ZnO	12	44	42

Table S1. RB5 adsorption percentage, RB5 maximum adsorption capacity, RB5 degradation percentage after 120 minutes of reaction for the synthesized ZnO materials and commercial ZnO. The experiments were performed at the natural pH of the dye, pH = 6.8.

The sample synthesized with ETG displayed the highest adsorption capacity (Q□$_{ax}$ = 85 mg/g), followed by ZnO-OA (80 mg/g) and bare ZnO (ZnO-SS, 76 mg/g). Lower capacities were found for ZnO-PVP (69 mg/g) and ZnO-CA (54 mg/g). Commercial ZnO exhibited the

lowest adsorption capacity among all samples, with a Q□ax of only 44 mg/g. As can be seen in the table, the higher the adsorption capacity of our synthesized materials, the higher the adsorption degree prior the degradation experiments, and the higher the dye degradation.

Mineralization degree of RB5

The mineralization degree of RB5 azo dye in the presence of ZnO nanoparticles, was measured by the total organic carbon (TOC) a Sievers InnovOx Total Organic Carbon analyzer. The percentage of mineralization was calculated using to the equation:

$$\% \, Mineralization = \left(1 - \frac{TOC_f}{TOC_i}\right) \times 100$$

where TOC_f and TOC_i are the total organic carbon concentration in the RB5 azo dye solution before and after the photocatalytic reaction, respectively.

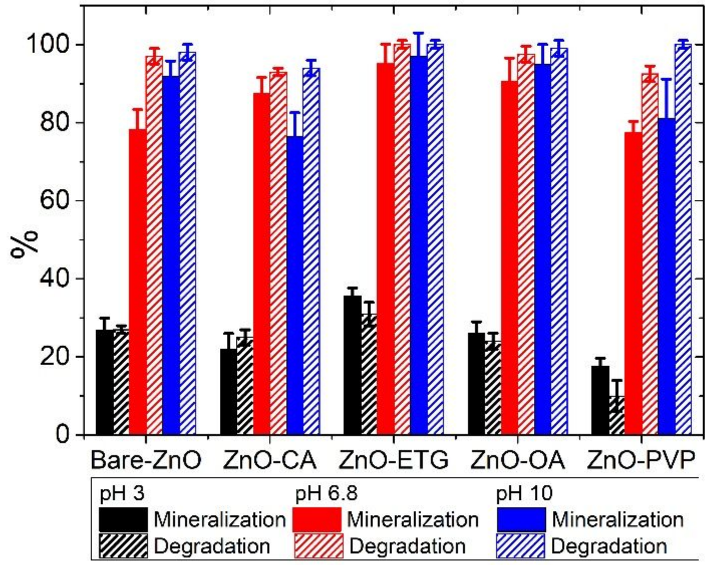

Fig. S7. Mineralization and degradation degree of the RB5 azo dye after the reaction process employing ZnO synthesized without capping agent (Bare-ZnO), CA, ETG, OA, and PVP.

Chemical structure of RB5 and MB

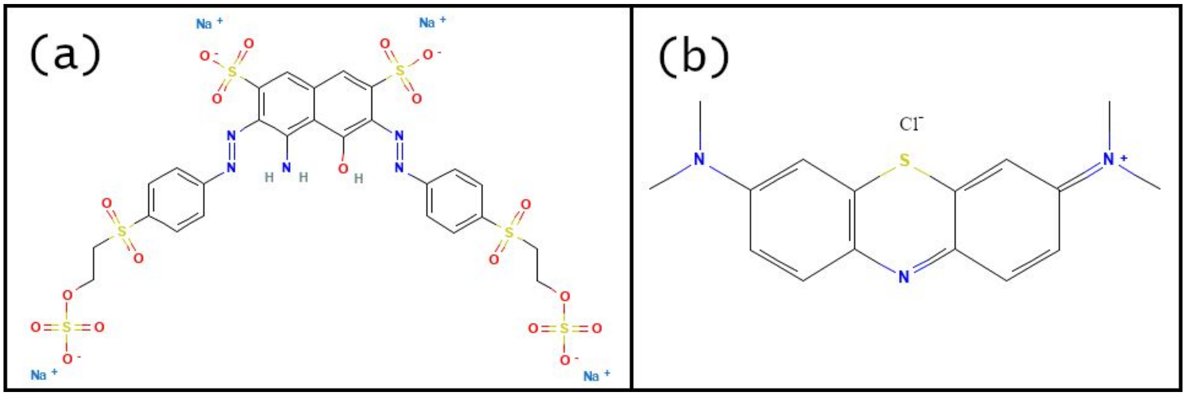

Fig. S8. Chemical structure of (a) RB5 dye and (b) MB dye.

Infrared spectra of ZnO NPs

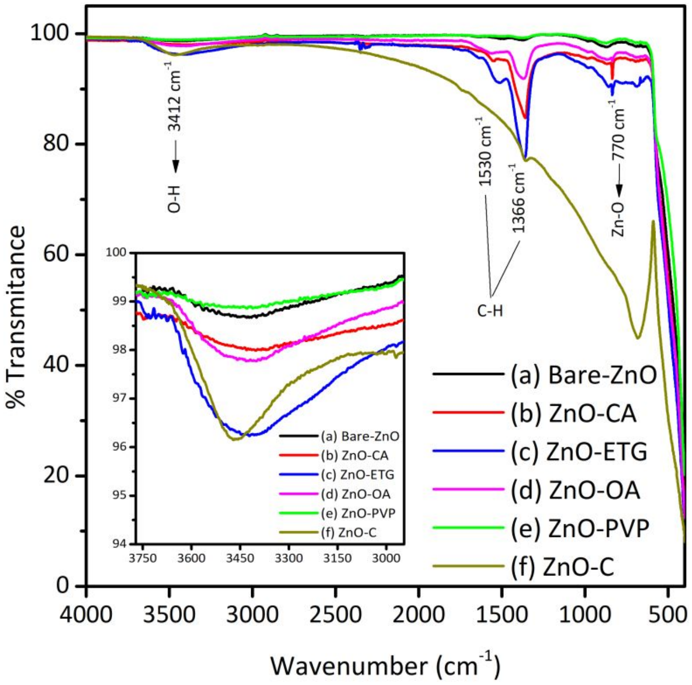

Fig. S9. Infrared spectra of the ZnO synthesized without and with capping agent; (a) Bare-ZnO, (b) ZnO-CA, (c) ZnO-ETG, (d) ZnO-OA, (e) ZnO-PVP, and (f) commercial ZnO.

All samples exhibit a prominent absorption band around 770 cm^{-1}, which corresponds to the stretching vibration of the Zn–O bond, confirming the successful formation of ZnO in all cases. A broad band centered at approximately 3412 cm^{-1} is observed in all spectra, and is attributed to the O–H stretching vibrations of surface hydroxyl groups and/or adsorbed water molecules. This band is particularly intense in the ZnO-ETG and ZnO-C samples.

Additional bands appear in the spectra of modified ZnO, indicating the presence of organic residues. Specifically, absorption bands at 1530 cm^{-1} and 1336 cm^{-1} are assigned to C=O and

C–H bending vibrations, respectively. These bands are especially evident in ZnO-CA, ZnO-ETG, and ZnO-OA, indicating partial retention of the capping molecules or their decomposition products. In contrast, the ZnO-PVP sample exhibits weaker organic-related bands, suggesting more effective removal of the capping agent after synthesis.

Raman spectra of ZnO NPs

Raman spectra were obtained using a Renishaw InVia Raman spectrometer with a 532 nm wavelength laser.

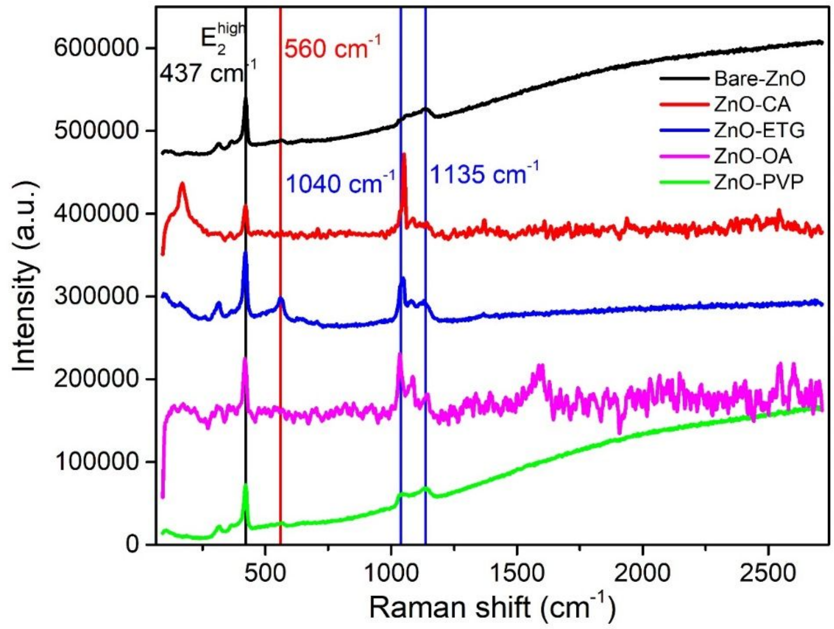

Fig. S10. Raman spectra of the ZnO synthesized without and with capping agent, as indicated in the legend, and commercial ZnO.

All samples display a prominent peak near 437 cm^{-1}, which corresponds to the E2(high) vibrational mode of oxygen atoms of hexagonal wurtzite ZnO. This confirms the formation of the wurtzite structure in all samples. Notably, ZnO-ETG, the sample with the highest photocatalytic activity (100% RB5 degradation), a distinct Raman band at around 560 cm^{-1}, associated with oxygen vacancies or lattice defects. This peak is much weaker or absent in the other samples, suggesting fewer active defect sites. In addition, a band between 1040 and 1140 cm^{-1} is observed in all organic modifiers-assisted samples (absent in Bare-ZnO), with the strongest intensity in ZnO-ETG. This band likely originates from residual organic species from the capping agent used in the synthesis. These residues may enhance photocatalytic activity by improving dye adsorption or altering surface charge dynamics.
